# The association between TyG and all-cause/non-cardiovascular mortality in general patients with type 2 diabetes mellitus is modified by age: results from the cohort study of NHANES 1999–2018

**DOI:** 10.1186/s12933-024-02120-6

**Published:** 2024-01-28

**Authors:** Younan Yao, Bo Wang, Tian Geng, Jiyan Chen, Wan Chen, Liwen Li

**Affiliations:** 1 Department of Cardiology, Guangdong Provincial People’s Hospital (Guangdong Academy of Medical Sciences), Southern Medical University, Guangzhou, 510080 China; 2 Guangdong Cardiovascular Institute, Guangdong Provincial People’s Hospital, Guangdong Academy of Medical Sciences, Guangzhou, 510080 China; 3 Department of Emergency Medicine, Research Center of Cardiovascular Disease, the People’s Hospital of Guangxi Zhuang Autonomous Region, Guangxi Academy of Medical Sciences, Nanning, 530021 China; 4 Department of Emergency Medicine, Guangdong Provincial People’s Hospital (Guangdong Academy of Medical Sciences), Southern Medical University, Guangzhou, 510080 China

**Keywords:** Triglyceride-glucose index, Insulin resistance, Type 2 diabetes mellitus, Cause-specific mortality, Age interaction

## Abstract

**Background:**

The prognostic value of triglyceride-glucose (TyG) index in general type 2 diabetes mellitus (T2DM) patients is still unclear. Therefore, we aimed to determine the associations between TyG and all-cause/cause-specific death in a T2DM cohort and explore whether such associations would be modified by age.

**Methods:**

A total of 3,376 patients with T2DM from the National Health and Nutrition Examination Survey (NHANES) 1999–2018 were selected and divided into the younger group (< 65 yrs) and the older group (≥ 65 yrs). Baseline TyG was calculated and cause-specific mortality status [cardiovascular (CV), cancer, and non-CV] was determined by the NHANES Public-Use Linked Mortality Files through 31 December 2019. Multivariate Cox and restricted cubic spline (RCS) regression models were used to evaluate the association between TyG and all-cause/cause-specific mortality. Interaction between TyG and age to mortality was also evaluated. Sensitivity analyses were performed in patients without cardiovascular disease, chronic kidney disease, or insulin treatment.

**Results:**

During a median follow-up of 107 months, 805 all-cause deaths occurred, of which 250 and 144 were attributed to CV and cancer deaths. There was a significant age interaction to the association between TyG and all-cause/non-CV mortality. After fully adjusting for potential confounding factors, higher TyG was associated with an increased risk of all-cause [TyG per unit increase Hazard Ratio (HR) 1.33, 95% Confidence Interval (CI) 1.06–1.66, *p* = 0.014] and non-CV mortality (TyG per unit increase HR 1.54, 95% CI 1.18–2.01, *p* = 0.002) only in the younger group, but not in the older group. There was no significant association between TyG and CV/cancer death in the total cohort and two age subgroups. Similar results were found in RCS and sensitivity analyses.

**Conclusion:**

In a national sample of patients with T2DM in the United States, we found that the association between TyG and all-cause/non-CV death was modified by age. Higher TyG was only associated with an increased risk of all-cause/non-CV only in T2DM patients younger than 65 years old, but not in older patients.

**Supplementary Information:**

The online version contains supplementary material available at 10.1186/s12933-024-02120-6.

## Background

The triglyceride-glucose (TyG) index had been developed and was shown to be a biochemical surrogate for identifying insulin resistance (IR) in individuals with and without diabetes [1, 2]. Different from other IR indices such as the homeostasis model assessment of insulin resistance (HOMA-IR) index, quantitative insulin sensitivity check index (QUICKI), and homeostasis model assessment of β-cell function (HOMA-β), TyG does not require insulin quantification and may be more useful to evaluate IR for T2DM patients regardless of insulin treatment. Recent studies have demonstrated that such a simple, easily available, and low-cost index is an independent predictor of future cardiovascular mortality, myocardial infarction, stroke, and incident type 2 diabetes mellitus (T2DM) in a general population, suggesting that TyG plays a role in predicting cardiovascular and metabolic diseases [3, 4]. Additionally, a series of studies also have confirmed TyG’s clinical value in predicting adverse cardiovascular events in nondiabetes or diabetes patients with baseline cardiovascular disease [5–8].

Emerging epidemiological researches showed that the large declines in all-cause mortality and cardiovascular (CV) mortality had led to non-cardiovascular (non-CV) death being the leading cause of death in T2DM patients [9, 10]. However, there is no study focused on TyG’s prognostic value of cause-specific death in general T2DM patients, and the results of prior studies with a small sample size showed that the prognostic value of TyG in T2DM patients were inconsistent [11–13]. Therefore, it is still unclear that whether the TyG index has prognostic value in general T2DM patients, especially in those without baseline cardiovascular disease.

It is also known that the serum glucose management for older patients with T2DM is sub-optimal [14, 15] because of potential risks of hypoglycemia and cognitive decline [16], which may further lead to adverse prognosis. In addition, some prior studies found that the prognostic value of TyG might be more prominent for young patients than old patients [17, 18]. As the TyG index is a formula composed of fasting triglyceride and glucose, we hypothesize that there is potential interaction between TyG and age to cause-specific mortality in general T2DM patients.

Therefore, in this study, we aimed to evaluate the associations between TyG and all-cause/cause-specific mortalities in a nationally representative sample of the United States (US) adults with T2DM and explore whether such associations would be modified by age. We also compared the prognostic value of TyG with other IR indices.

## Methods

### Study population

The National Health and Nutrition Examination Survey (NHANES) is an ongoing program of study that provides population estimates related to nutrition and health of adults and children in the US [19]. In 1999, the survey became a continuous program focusing on a variety of health and nutrition measurements to meet emerging needs. The survey used a stratified, multistage probability design to recruit a representative sample of the US population. Data were obtained via personal structured interviews at home, health examinations at a mobile examination center, and specimen analyses in the laboratory [19].

**Fig. 1:**
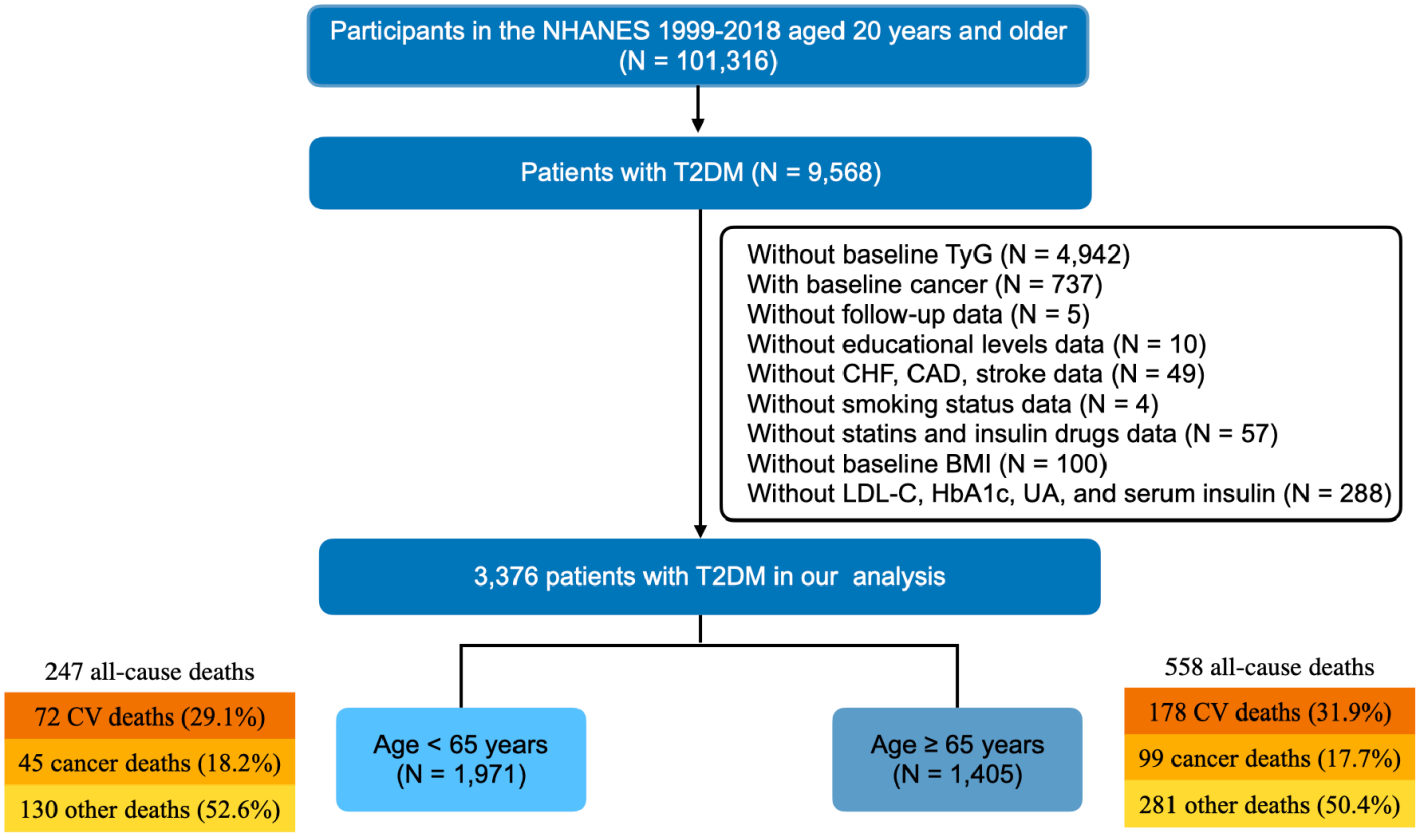
Flowchart of the study. BMI, body mass index; CAD, coronary artery disease; CHF, chronic heart failure; CV, cardiovascular; HbA1c, glucated hemoglobin; LDL-C, low-density-lipoprotein cholesterol; T2DM, type 2 diabetes mellitus; TyG, triglyceride-glucose; UA, uric acid

We used the data from NHANES 1999–2018. Patients with T2DM aged ≥ 20 years at enrollment were selected. T2DM was defined as self-reported doctor diagnosis of diabetes, use of insulin or oral glucose lowering drugs, plasma fasting glucose levels ≥ 7.0 mmol/L (126 mg/dL), random blood glucose or 2 h oral glucose tolerance test blood glucose ≥ 11.1 mmol/L (200 mg/dL), or glycated hemoglobin A1c (HbA1c) ≥ 6.5%. We excluded participants who: (1) without baseline TyG; (2) had any cancer at baseline; (3) without follow-up data; (4) without important baseline clinical measurements. In the end, a total of 3,376 patients with T2DM were included in this study (Fig. 1).

### Insulin resistance indices

Four insulin resistance indices were calculated according to previous studies [1, 20, 21]: (1) TyG index = Ln [fasting triglyceride (mg/dL) × fasting glucose (mg/dL)/2]; (2) HOMA-IR index = fasting glucose (mmol/L) × fasting insulin (µU/mL)]/22.5; (3) QUICKI index = 1/[log(fasting insulin, µU/mL) + log(fasting plasma glucose, mg/dL)]; (4) HOMA-β index = 20 × fasting insulin (µIU/ml)/[fasting glucose (mmol/ml) − 3.5].

### Assessment of covariates

Standardized questionnaires obtained information on age, gender, ethnicity, educational level, smoking status, weight, height, presence of hypertension, coronary artery disease (CAD), stroke, congestive heart failure (CHF), drug use (including insulin, oral glucose lowering drugs, antihypertensive drugs, aspirin, and statin). Educational levels were classified as less than high school, high school or equivalent, and college or above. Smoking statuses were classified as current smoking or not. Body mass index (BMI) = weight (kg)/height (m)^2^. Hypertension was defined as having a self-reported history of hypertension or use of antihypertensive drug or systolic blood pressure ≥ 140 mmHg or diastolic pressure blood ≥ 90 mmHg. CAD was defined as self-reported coronary heart disease or myocardial infarction. Chronic kidney disease (CKD) was defined as estimated glomerular filtration rate (eGFR) < 60 mL/(1.73m^2^*min). Estimated glomerular filtration rate was calculated from serum creatinine using the CKD-EPI Eq. [22]. Random urine albumin-creatinine ratio (UACR) was calculated by dividing the urinary albumin (mg/dL) concentration by the urinary creatinine concentration (mg/L). Antihypertensive drugs included angiotensin converting enzyme inhibitors, angiotensin II receptor blockers, diuretic, calcium channel blockers, β-blockers, adrenergic blockers, and aldosterone receptor antagonists. Oral glucose lowering drugs included biguanides, sulfonylureas, thiazolidinediones, dipeptidyl peptidase 4 inhibitors, GLP-1 receptor agonists, sodium-glucose co-transporter protein 2 inhibitors, alpha-glucosidase inhibitors, or glinides.

In addition, strict laboratory analyses were performed, including the assessment of fasting glucose, fasting triglycerides, fasting insulin, HbA1c, total cholesterol, low density lipoprotein cholesterol (LDL-C), high density lipoprotein cholesterol (HDL-C), serum albumin, serum creatinine, urinary albumin, urinary creatinine, and uric acid (UA) at baseline. Further details of these measurements were documented in the NHANES Laboratory Medical Technologists Procedures Manual [19].

### Ascertainment of mortality

To determine the mortality status of patients during follow-up, we used the NHANES Public-Use Linked Mortality Files which were linked to the National Death Index records by a probability matching algorithm through 31 December 2019 [23]. All-cause mortality was defined as death from any cause. Specific causes of death were determined using the International Statistical Classification of Disease, 10th Revision (ICD-10). CV death included rheumatic heart diseases, hypertensive heart and renal disease, ischemic heart disease, and heart failure (ICD-10 codes I00–I09, I11, I13, and I20–I51). Cancer death was defined as death from malignant neoplasms (ICD-10 codes C00-C97). Except cardiovascular cause, other patients who died were attributed to non-CV death. Detailed information about causes of death was displayed in Supplemental Table 1.

### Statistical analysis

All patients were divided into the younger group (age < 65 years) and the older group (age ≥ 65 years). Baseline characteristics were reported as median (25th-75th percentile) for continuous variables and number (percentage) for categorical variables, and compared between age groups. We also compared the baseline characteristics across TyG groups according to tertiles. Differences among groups were compared using the Wilcoxon-rank test, Kruskal-Wallis test, or chi-square test as appropriate. Correlations of TyG with other IR indices were assessed using the Spearman correlation test.

To evaluate the association between TyG and all-cause/cause-specific mortality, the TyG index was analyzed as both a continuous and a categorical variable. Cox proportional hazards analyses were performed to compute hazard ratios (HRs) and 95% confidence intervals (CIs). The proportional hazards assumption was tested by inspection of Schoenfeld residuals. Models for univariate and multivariate analyses were built and age interaction was assessed: Model 1 was unadjusted; Model 2 was adjusted for age and gender; Model 3 was adjusted for covariates selected by the backward stepwise method in terms of Akaike Information Criterion; Model 4 was fully adjusted for covariates including age, gender, ethnicity, educational level, current smoking, BMI, hypertension, CAD, CHF, albumin, LDL-C, HbA1c, eGFR, UACR, UA, insulin drug, metformin, and statin. To further explore the potential nonlinear association between TyG index and mortality, we used the restricted cubic spline (RCS) regression model with three knots (10%, 50%, and 90%).

To compared the prognostic value of TyG with other IR indices (as well as fasting glucose and triglyceride alone), we introduced each indices into a base model (with covariates in Model 3) respectively. C-indices of models with different indices were compared to evaluate the difference of discrimination properties.

Sensitivity analyses were performed in patients without CHF, without CAD, without stroke, without CKD, and without insulin treatment.

All analyses were performed using R software version 4.0.1. Due to the exploratory nature of these analyses, no adjustments were made for multiple comparisons. *P* < 0.05 were considered statistically significant.

## Results

### Baseline characteristics

The baseline characteristics of the patients by age groups were summarized in Table 1. Among the 3,376 participants, the median age was 62 years, 1,750 (51.84%) were male, and the median TyG was 9.10. There were significant differences in ethnic composition, educational levels, and smoking status between two age groups. Compared with the older patients with T2DM, younger patients had higher TyG, HOMA-IR, and HOMA-β, but lower QUICKI, which indicated that young patients had more severe IR status. Patients in the older group had more comorbidities such as CHF, CAD, hypertension, CKD, and stroke than those in the younger group. However, the levels of BMI, HbA1c, glucose, triglyceride, serum insulin, total cholesterol, LDL-C were lower but serum creatinine, UACR, UA, and HDL-C were higher in the older group. As for the medication treatments, older patients received more glucose lowering drugs, antihypertensive drugs, statin, and aspirin. The baseline characteristics of the patients by TyG tertiles were displayed in Supplemental Table 2.

**Table 1:** Baseline characteristics of patients with T2DM by age

Variables	Total (N = 3,376)	< 65 yrs (N = 1,971)	≥ 65 yrs (N = 1,405)	P value
Age (yrs)	62 (51, 70)	53 (45, 60)	72 (68, 78)	< 0.001
Male	1,750 (51.84%)	1,015 (51.50%)	735 (52.31%)	0.665
BMI (kg/m^2^)	30.7 (26.7, 35.8)	31.9 (27.5, 37.6)	29.3 (25.9, 33.1)	< 0.001
**Ethnicity**				< 0.001
Mexican American	692 (20.5%)	443 (22.5%)	249 (17.7%)	
Non-Hispanic Black	816 (24.2%)	523 (26.5%)	293 (20.9%	
Non-Hispanic White	1,166 (34.5%)	560 (28.4%)	606 (43.1%)	
Other Hispanic	358 (10.6%)	230 (11.7%)	128 (9.1%)	
Other Race	344 (10.2%)	215 (10.9%)	129 (9.2%)	
**Educational level**				< 0.001
Less than high school	1229 (36.40%)	628 (31.86%)	601 (42.78%)	
High school or equivalent	787 (23.31%)	466 (23.64%)	321 (22.85%)	
College or above	1360 (40.28%)	877 (44.50%)	483 (34.38%)	
Current smoking	587 (17.39%)	459 (23.29%)	128 (9.11%)	< 0.001
**Comorbidities**				
Congestive heart failure	263 (7.79%)	88 (4.46%)	175 (12.46%)	< 0.001
Coronary artery disease	479 (14.19%)	184 (9.34%)	295 (21.00%)	< 0.001
Hypertension	2678 (79.32%)	1393 (70.67%)	1285 (91.46%)	< 0.001
Stroke	239 (7.08%)	85 (4.31%)	154 (10.96%)	< 0.001
CKD	541 (16.0%)	105 (5.3%)	436 (31.0%)	< 0.001
**IR indices**				
TyG	9.10 (8.67, 9.55)	9.15 (8.72, 9.65)	9.02 (8.58, 9.45)	< 0.001
TyG group				< 0.001
< 8.82	1,124 (33.29%)	599 (30.39%)	525 (37.37%)	
8.82–9.37	1,124 (33.29%)	639 (32.42%)	485 (34.52%)	
> 9.37	1,128 (33.41%)	733 (37.19%)	395 (28.11%)	
HOMA-IR	4.84 (2.73, 8.42)	5.50 (3.11, 9.42)	4.15 (2.33, 6.92)	< 0.001
QUICKI	0.30 (0.28, 0.33)	0.30 (0.28, 0.32)	0.31 (0.29, 0.34)	< 0.001
HOMA-β	70.09 (37.85, 123.04)	75.14 (37.66, 134.35)	65.06 (38.56, 109.67)	0.002
**Laboratory measurements**				
HbA1c (%)	6.6 (6.0, 7.7)	6.7 (6.0, 8.1)	6.5 (6.0, 7.3)	< 0.001
Glucose (mg/dL)	133 (115, 167)	136 (116, 178)	131 (114, 157)	< 0.001
Triglyceride (mg/dL)	127 (90, 184)	131 (93, 193)	123 (86, 175)	< 0.001
Insulin (uU/mL)	13.7 (8.3, 22.9)	15.2 (8.9, 25.2)	12.1 (7.6, 19.8)	< 0.001
Albumin (g/dL)	4.1 (3.9, 4.4)	4.1 (3.9, 4.4)	4.1 (3.9, 4.4)	0.909
Serum creatinine (umol/L)	76 (62, 92)	71 (59, 84)	84 (71, 106)	< 0.001
eGFR (mL/1.73m^2^*min)	89 (69, 103)	99 (85, 110)	72 (56, 87)	< 0.001
UACR (mg/g)	12.4 (6.6, 41.9)	11.0 (5.9, 31.1)	16.2 (7.6, 57.6)	< 0.001
UACR category				< 0.001
<10 mg/g	1397 (41.38%)	904 (45.87%)	493 (35.09%)	
10 ~ < 30 mg/g	973 (28.82%)	563 (28.56%)	410 (29.18%)	
30 ~ < 300 mg/g	766 (22.69%)	370 (18.77%)	396 (28.19%)	
≥ 300 mg/g	240 (7.11%)	134 (6.80%)	106 (7.54%)	
Uric acid (umol/L)	339.0 (279.6, 398.5)	333.1 (273.6, 392.6)	350.9 (297.4, 410.4)	< 0.001
Total cholesterol (mmol/L)	4.8 (4.1, 5.5)	4.9 (4.2, 5.6)	4.6 (3.9, 5.4)	< 0.001
HDL-C (mmol/L)	1.2 (1.0, 1.5)	1.2 (1.0, 1.5)	1.3 (1.1, 1.6)	< 0.001
LDL-C (mmol/L)	2.7 (2.1, 3.4)	2.9 (2.3, 3.5)	2.5 (1.9, 3.2)	< 0.001
**Medications**				
Glucose lowering drug	1,902 (56.34%)	1,073 (54.44%)	829 (59.00%)	0.009
Insulin drug	257 (7.61%)	143 (7.26%)	114 (8.11%)	0.389
Metformin	924 (27.37%)	567 (28.77%)	357 (25.41%)	0.034
Anti-hypertension drug	2,144 (63.51%)	1,025 (52.00%)	1,119 (79.64%)	< 0.001
Statins	1,385 (41.02%)	654 (33.18%)	731 (52.03%)	< 0.001
Aspirin	122 (3.61%)	48 (2.44%)	74 (5.27%)	< 0.001
Fibrates	46 (1.36%)	28 (1.42%)	18 (1.28%)	0.846

Values are number (percentage), or median (25th-75th percentile)

BMI, body mass index; CKD, chronic kidney disease; eGFR, estimated glomerular filtration rate; HbA1c, glucated hemoglobin; HDL-C, high-density-lipoprotein cholesterol; HOMA, homeostasis model assessment; IR, insulin resistance; LDL-C, low-density-lipoprotein cholesterol; QUICKI, quantitative insulin sensitivity check index; UACR, urine albumin-creatinine ratio

**Fig. 2:**
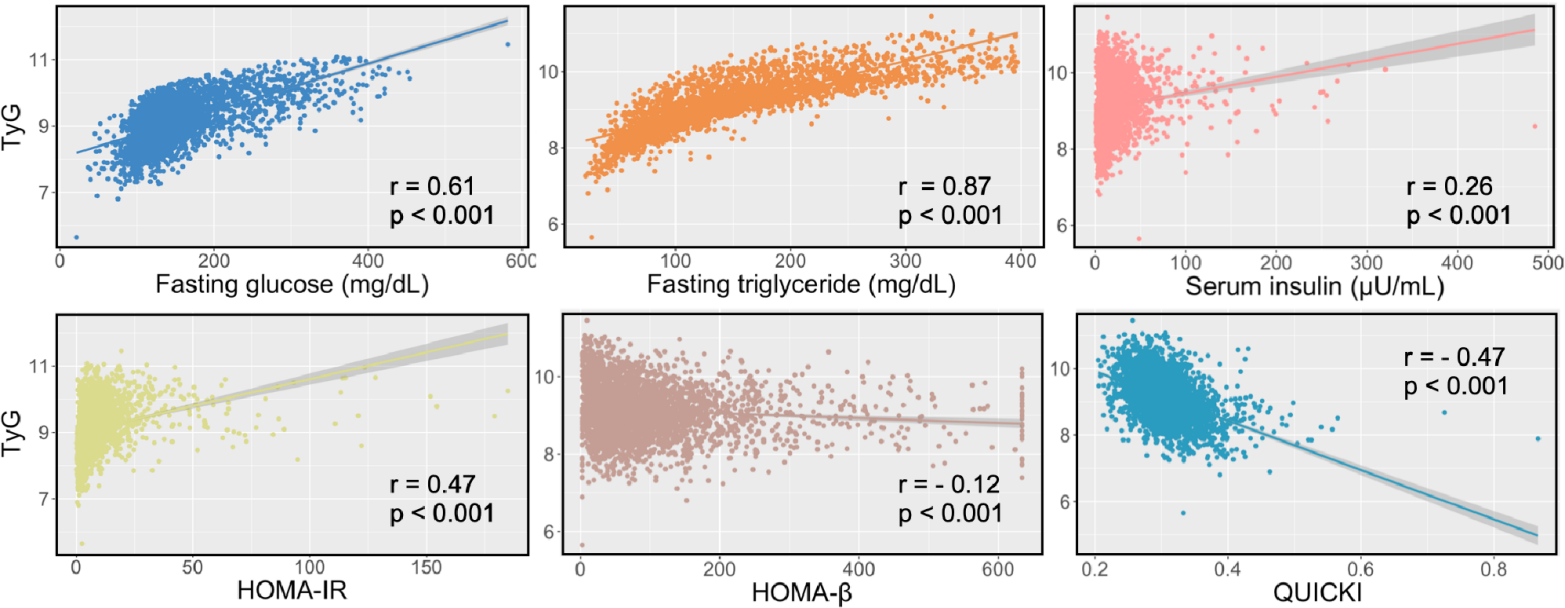
Relationship between TyG and other insulin resistance indices. HOMA-IR, homeostasis model assessment of insulin resistance; QUICKI, quantitative insulin sensitivity check index; HOMA-β, homeostasis model assessment of β-cell function

Figure 2 showed that TyG had significant positive correlations with fasting triglyceride (*r* = 0.87), fasting glucose (*r* = 0.61), HOMA-IR (*r* = 0.47), and serum insulin (*r* = 0.26). While negative correlations were observed between TyG and HOMA-β (*r* = -0.12) as well as QUICKI (*r* = -0.47). When we analyzed in age subgroups, most correlations were also statistically significant, except HOMA-β (*r* =-0.02, *p* = 0.566) in older patients (Supplemental Fig. 1).

### Cause-specific death and TyG in T2DM

During a median follow-up of 107 months, 805 (23.8%) all-cause deaths occurred, of which 250 and 144 were attributed to CV and cancer deaths. Non-CV death accounted for 68.9% of all-cause deaths in the total cohort. The compositions of cause-specific death were similar between two age groups.

In univariable Cox analysis, there was no association between TyG (as a continuous variable) and all-cause mortality in the total cohort. However, TyG as a categorical variable was associated with all-cause mortality and patients with TyG ranging from 8.82 to 9.37 had a lower risk (HR 0.79, 95% CI: 0.66–0.94, *p* < 0.001) compared with those with TyG < 8.82 (Table 2). The p value for age interaction was statistically significant (p for interaction < 0.001). In the younger group, TyG (as both continuous and categorical variables) was associated with all-cause mortality (TyG per unit increased: HR 1.47, 95% CI 1.22–1.76, *p* < 0.001; TyG < 8.82 as reference, 8.82 ≤ TyG ≤ 9.37 h 0.96, 95%CI 0.67–1.38, *p* = 0.840, TyG > 9.37, HR 1.57, 95% CI 1.15–2.14, *p* = 0.005); while in the older group, only patients with TyG ranging from 8.82 to 9.37 had a lower risk. In Model 2 which adjusted for age and sex, similar associations between TyG and all-cause mortality were observed (Table 2). After fully adjusting for potential confounders (Model 3 and Model 4), the age interaction was still statistically significant and the TyG was only significantly associated with all-cause mortality in patients of the younger group (Table 2).

**Table 2:** Association between TyG and all-cause mortality

	Hazard ratio (95% confidence interval)			
	Model 1	Model 2	Model 3	Model 4
TyG (continuous)	P for interaction < 0.001	P for interaction < 0.001	P for interaction < 0.001	P for interaction < 0.001
Total	0.98 (0.88–1.09)	1.10 (0.98–1.23)	1.11 (0.99–1.25)	1.00 (0.88–1.14)
< 65 yrs	1.47 (1.22–1.76)* **P < 0.001**	1.50 (1.24–1.81)* **P < 0.001**	1.48 (1.23–1.79)* **P < 0.001**	1.33 (1.06–1.66)* **P = 0.014**
≥ 65 yrs	0.89 (0.77–1.02)	0.94 (0.81–1.08)	0.98 (0.85–1.13)	0.91 (0.78–1.12)
TyG (categorical)	P for interaction < 0.001	P for interaction < 0.001	P for interaction < 0.001	P for interaction < 0.001
TyG tertile 1	Reference	Reference	Reference	Reference
Total				
TyG tertile 2	0.79 (0.66–0.94)* **P = 0.008**	0.80 (0.67–0.96)* **P = 0.014**	0.89 (0.74–1.06)	0.83 (0.69–0.99)* **P = 0.043**
TyG tertile 3	0.92 (0.78–1.08)	1.06 (0.90–1.26)	1.12 (0.94–1.33)	0.96 (0.79–1.16)
< 65 yrs				
TyG tertile 2	0.96 (0.67–1.38)	0.92 (0.64–1.31)	1.02 (0.71–1.47)	0.97 (0.67–1.40)
TyG tertile 3	1.57 (1.15–2.14)* **P = 0.005**	1.56 (1.14–2.14)* **P = 0.005**	1.66 (1.20–2.30)* **P = 0.002**	1.40 (1.03–1.99)* **P = 0.046**
≥ 65 yrs				
TyG tertile 2	0.71 (0.58–0.87)* **P < 0.001**	0.81 (0.66–0.99)* **P < 0.043**	0.90 (0.73–1.10)	0.84 (0.68–1.04)
TyG tertile 3	0.83 (0.67–1.01)	0.90 (0.74–1.11)	0.96 (0.78–1.19)	0.87 (0.69–1.09)

Model 1 was unadjusted

Model 2 was adjusted for age and gender

Model 3 was adjusted for age, gender, ethnicity, BMI, CHF, CAD, albumin, LDL-C, eGFR, UACR, Insulin treatment, and metformin

Model 4 was adjusted for age, gender, ethnicity, educational level, current smoking, BMI, CHF, CAD, hypertension, albumin, LDL-C, eGFR, HbA1c, UACR, UA, Insulin treatment, metformin, and statins

*indicated that the p value was less than 0.05

As for non-CV death, results of the multivariate analyses did not show any significant association between TyG and non-CV death in the total cohort, but significant age interaction was observed (all p for interaction ≤ 0.001). Age subgroup analyses indicated that a higher TyG was associated with an increased risk of non-CV mortality only in patients of the younger group (Model 4: TyG per unit increased HR 1.54, 95% CI 1.18–2.01, *p* < 0.001; TyG < 8.82 as reference, 8.82 ≤ TyG ≤ 9.37 h 0.93, 95%CI 0.60–1.45, *p* = 0.8 = 751, TyG > 9.37, HR 1.44, 95% CI 1.01–2.18, *p* = 0.044). Details were displayed in Table 3.

**Table 3:** Association between TyG and non-cardiovascular mortality

	Hazard ratio (95% confidence interval)			
	Model 1	Model 2	Model 3	Model 4
TyG (continuous)	P for interaction < 0.001	P for interaction < 0.001	P for interaction < 0.001	P for interaction < 0.001
Total	0.98 (0.86–1.11)	1.09 (0.95–1.25)	1.09 (0.95–1.25)	1.05 (0.90–1.22)
< 65 yrs	1.55 (1.25–1.94)* **P < 0.001**	1.59 (1.27-2.00)* **P < 0.001**	1.60 (1.28-2.00)* **P < 0.001**	1.54 (1.18–2.01)* **P < 0.002**
≥ 65 yrs	0.85 (0.72–1.01)	0.90 (0.76–1.06)	0.91 (0.77–1.08)	0.92 (0.76–1.12)
TyG (categorical)	P for interaction = 0.002	P for interaction = 0.002	P for interaction < 0.001	P for interaction = 0.001
TyG tertile 1	Reference	Reference	Reference	Reference
Total				
TyG tertile 2	0.79 (0.64–0.98)* **P = 0.032**	0.81 (0.65–0.99)* **P = 0.045**	0.87 (0.70–1.07)	0.84 (0.68–1.05)
TyG tertile 3	0.92 (0.75–1.12)	1.06 (0.87–1.30)	1.07 (0.87–1.32)	1.01 (0.81–1.27)
< 65 yrs				
TyG tertile 2	0.94 (0.61–1.43)	0.89 (0.58–1.36)	0.98 (0.63–1.51)	0.93 (0.60–1.45)
TyG tertile 3	1.55 (1.07–2.24)* **P = 0.020**	1.54 (1.07–2.24)* **P = 0.021**	1.64 (1.12–2.41)* **P = 0.011**	1.44 (1.01–2.18)* **P = 0.048**
≥ 65 yrs				
TyG tertile 2	0.72 (0.56–0.92)* **P = 0.008**	0.82 (0.64–1.04)	0.87 (0.68–1.12)	0.87 (0.67–1.12)
TyG tertile 3	0.82 (0.64–1.05)	0.90 (0.70–1.15)	0.92 (0.71–1.18)	0.94 (0.71–1.34)

Model 1 was unadjusted

Model 2 was adjusted for age and gender

Model 3 was adjusted for age, gender, ethnicity, educational level, BMI, CHF, albumin, eGFR, UACR, insulin treatment, and metformin

Model 4 was adjusted for age, gender, ethnicity, educational level, current smoking, BMI, CHF, CAD, hypertension, albumin, LDL-C, eGFR, HbA1c, UACR, UA, Insulin treatment, metformin, and statins

*indicated that the p value was less than 0.05

We did not observe any significant association between TyG and CV/cancer death in the total cohort and two age groups after adjustments (Supplemental Tables 3 and 4).

### RSC regression analysis

**Fig. 3:**
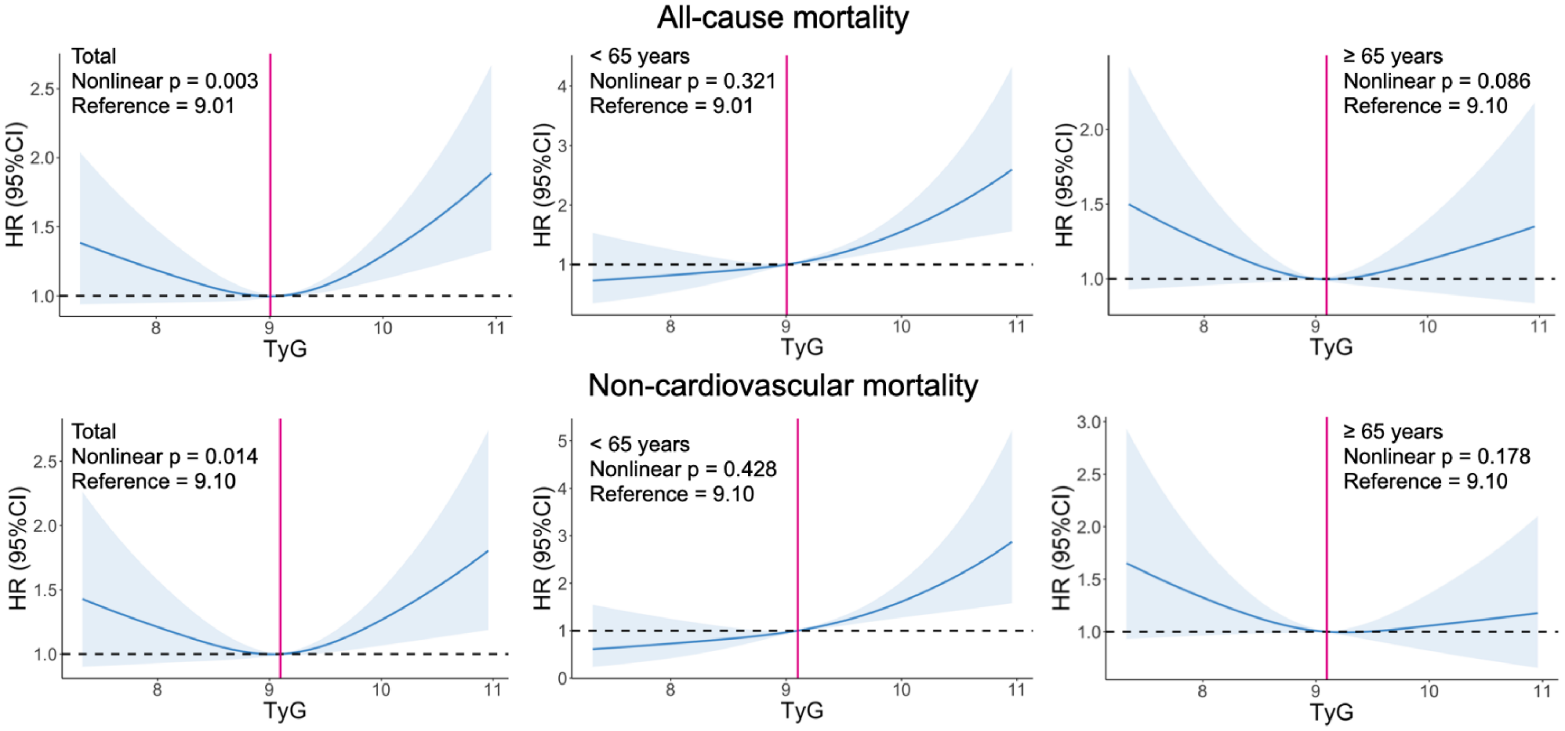
Association between TyG and all-cause/non-cardiovascular mortality. Models for all-cause mortality were adjusted for age, gender, ethnicity, BMI, CHF, CAD, albumin, LDL-C, eGFR, UACR, Insulin treatment, and metformin. In total cohort, compared with a TyG of 9.01, patients with a TyG ≥ 9.32 had a higher risk of all-cause mortality; there was a linear association between TyG and all-cause death in the younger group; while there was no significant association between TyG and all-cause death in the older group. Models for non-cardiovascular mortality were adjusted for age, gender, ethnicity, educational level, BMI, CHF, albumin, eGFR, insulin treatment, and metformin. In total cohort, compared with a TyG of 9.10, patients with a TyG ≥ 9.48 had a higher risk of non-cardiovascular mortality; there was a linear association between TyG and non-cardiovascular death in the younger group; while there was no significant association between TyG and non-cardiovascular death in the older group

In the total cohort, there was a J-shaped association between TyG and all-cause/non-CV mortality (Fig. 3) and the p value of age interaction was < 0.001. Compared with patients with TyG of 9.01, those with a TyG ≥ 9.32 had a higher risk of all-cause mortality; in the younger group, a linear association between TyG and all-cause death was observed, while there was no significant association between TyG and all-cause death in the older group. As for non-CV mortality, similar linear association was also observed in the younger group (Fig. 3). While there were no significant relationships between TyG and CV/cancer death (Supplemental Fig. 2).

### Predictive power of models with different IR indices

Table 4 showed the predictive power of various models with different IR indices. The C-indices of models with HOMA-IR, QUICKI, and HOMA-bata were not significant different from which of the model with TyG. However, the C-indices of models with TyG for all-cause/non-CV deaths were significantly higher than those with fasting triglyceride in the younger group. And the performance of TyG in predicting non-CV death was also better than which of fasting glucose in younger patients. Similar results were observed when we excluded patients with insulin treatments (Supplemental Table 5).

**Table 4:** Predictive power of various models with different IR indices (Reference = TyG model)

Base model	+ TyG	+ HOMA-IR		+ QUICKI		+ HOMA-beta		+ Glucose		+ Triglyceride	
	C-index (95% CI)	C-index (95% CI)	P value	C-index (95% CI)	P value	C-index (95% CI)	P value	C-index (95% CI)	P value	C-index (95% CI)	P value
All-cause mortality											
Total	0.80 (0.78–0.82)	0.80 (0.78–0.82)	0.477	0.80 (0.78–0.81)	0.151	0.80 (0.78–0.81)	0.302	0.80 (0.78–0.82)	0.646	0.80 (0.78–0.82)	0.356
< 65 yrs	0.76 (0.73–0.79)	0.75 (0.72–0.79)	0.204	0.75 (0.71–0.78)	0.119	0.75 (0.71–0.78)	0.073	0.76 (0.73–0.79)	0.838	0.75 (0.72–0.79)	**0.021**
≥ 65 yrs	0.75 (0.73–0.77)	(0.75 (0.73–0.77)	0.909	0.75 (0.73–0.77)	0.726	0.75 (0.73–0.77)	0.658	0.75 (0.73–0.77)	0.972	0.75 (0.73–0.77)	0.775
Cardiovascular mortality											
Total	0.82 (0.78–0.85)	0.82 (0.80–0.85)	0.982	0.82 (0.80–0.85)	0.911	0.82 (0.80–0.85)	0.882	0.82 (0.80–0.85)	0.816	0.82 (0.79–0.85)	0.404
< 65 yrs	0.82 (0.76–0.88)	0.82 (0.75–0.88)	0.788	0.82 (0.75–0.88)	0.736	0.81 (0.75–0.88)	0.626	0.82 (0.77–0.88)	0.496	0.81 (0.75–0.87)	0.579
≥ 65 yrs	0.77 (0.73–0.81)	0.78 (0.74–0.81)	0.298	0.78 (0.74–0.81)	0.319	0.78 (0.74–0.81)	0.472	0.77 (0.73–0.81)	0.938	0.77 (0.73–0.81)	0.802
Cancer mortality											
Total	0.75 (0.71–0.79)	0.75 (0.71–0.79)	0.400	0.75 (0.71–0.79)	0.837	0.75 (0.71–0.79)	0.721	0.75 (0.71–0.79)	0.563	0.75 (0.71–0.79)	0.441
< 65 yrs	0.70 (0.62–0.78)	0.70 (0.62–0.78)	0.966	0.70 (0.62–0.77)	0.793	0.69 (0.62–0.77)	0.623	0.69 (0.62–0.77)	0.831	0.71 (0.63–0.79)	0.446
≥ 65 yrs	0.64 (0.58–0.70)	0.64 (0.58–0.71)	0.933	0.65 (0.59–0.71)	0.834	0.65 (0.58–0.71)	0.854	0.65 (0.58–0.71)	0.760	0.64 (0.58–0.70)	0.949
Non-cardiovascular mortality											
Total	0.79 (0.77–0.81)	0.79 (0.77–0.81)	0.160	0.79 (0.77–0.81)	0.241	0.79 (0.77–0.81)	0.275	0.79 (0.77–0.81)	0.159	0.79 (0.77–0.81)	0.389
< 65 yrs	0.75 (0.72–0.79)	0.74 (0.70–0.78)	0.598	0.74 (0.70–0.78)	0.428	0.73 (0.69–0.77)	0.177	0.73 (0.69–0.78)	**0.050**	0.73 (0.70–0.77)	**0.043**
≥ 65 yrs	0.74 (0.72–0.77)	0.74 (0.72–0.77)	0.700	0.74 (0.72–0.77)	0.602	0.74 (0.72–0.77)	0.698	0.74 (0.72–0.77)	0.643	0.74 (0.72–0.77)	0.853

Base models included the covariates which were selected by the stepwise method in Model 3

### Sensitivity analysis

Sensitivity analyses showed that most results of the associations between TyG and all-cause/cause-specific mortality were robust in T2DM patients without CHF, without CAD, without stroke, without CKD, or without insulin treatment at baseline (Supplemental Tables 6–9).

## Discussion

Our study explored the associations between TyG and all-cause/cause-specific deaths in a T2DM population selected from the NHANES cohort and compared the prognostic value of TyG with other IR indices. We found that the associations between TyG and all-cause/non-CV death in general T2DM patients were modified by age. Higher TyG was only associated with an increased risk of all-cause/non-CV mortality only in T2DM patients younger than 65 years old, but not in the older patients. There were no significant associations between TyG and CV/cancer mortality in general T2DM patients. Other IR indices including HOMA-IR, QUICKI, and HOMA-bata did not show better prognostic value than TyG based on the models with traditional risk factors. The results of the sensitivity analyses suggested that our findings were robust.

Previous studies showed that higher TyG was associated with an increased risk of adverse events in patients with CV disease [7, 8, 13, 24], which indicated that TyG was a useful predictor of prognosis for patients with CV disease. However, the predictive value of TyG in T2DM patients was unclear. A prior study based on the Medical Information Mart for Intensive Care III database found that TyG was not associated with intensive care unit death or in-hospital death in patients with T2DM [11]. By contrast, a study including 555 patients with diabetic foot ulcers implied a strong positive correlation between the TyG index and all-cause mortality [12]. Another recent study also found that a higher TyG associated with an increased risk of all-cause mortality in 231 patients with acute coronary syndrome and T2DM aged ≥ 80 years [13]. While these two studies were all with a small sample size and most patients had CV disease.

Our study included 3,376 general T2DM patients from a national representative sample of the US population to fill the knowledge gap that whether TyG was associated with all-cause/cause-specific mortality in general T2DM patients. We found that TyG was associated with all-cause death but not CV death. In addition, our study also explored the prognostic value of TyG for cancer and non-CV mortality in T2DM patients. Recent epidemiological studies found that the large decline in vascular disease death rates led to a transition from vascular causes to non-CV cause as the leading contributor to death rates in individuals with T2DM [9, 10]. In our study, we found that the leading cause of death was non-CV cause (accounting for more than 2/3) and there was a positive association between TyG and non-CV mortality in young T2DM patients.

It was notable that the results of our study showed that the associations between TyG and all-cause/non-CV mortality were modified by age (only significant in patients with T2DM younger than 65 years old). Although young patients had less cardiovascular diseases at baseline, lower proportion of glucose lowering drug treatment and higher levels of blood glucose and HbA1c indicated that young patients had worse blood glucose control than old patients in our study. These findings were similar to those in a previous study that control of glycaemia and cardiovascular risk factors was better among old T2DM patients than young patients [25]. Notably, when excluding patients with CV diseases at baseline in sensitivity analyses, the results were similar which suggested that the prognostic value of TyG in young T2DM patients was not attributed to those with baseline CV diseases. These findings confirmed the prognostic value of TyG for young T2DM patients which would be helpful to identify those at high risk of all-cause/non-CV death.

It was surprising that TyG was not associated with all-cause/cause-specific deaths in the older patients and similar results were observed in those without baseline CV diseases in our study. Compared with previous studies [11–13], the strengths of our study were a larger sample size, longer follow-up, and more deaths, which allowed us to eliminate more potential influences of confounding factors on the results of multivariate analysis. Additionally, patients in our cohort had better glycaemia control (lower levels of HbA1c and fasting blood glucose) and a relative low risk of CV events (lower LDL-C and less smoking) than those in previous studies [12, 13, 26]. There were two possible explanations for such an age difference: (1) Compared with young patients, old patients had more comorbidities and worse organ function, and these factors might be of higher prognostic value of death than TyG index (without additional prognostic value) for old T2DM patients rather than young patients. (2) The better glucose and triglyceride control at baseline in the older group in our study indicated that old patients might pay more attention to their serum glucose and triglyceride levels than young patients. Those with higher TyG at baseline might receive intensive treatment during follow-up, which would optimize fasting glucose and triglyceride and consequently reduce the prognostic value of baseline TyG. Therefore, we suggest that TyG has no prognostic value of predicting death in old T2DM patients with good glycaemia control and low baseline CV risks. However, a post-hoc analysis of the Action to Control Cardiovascular Risk in Diabetes trial found that the cumulative TyG index independently predicts the incidence of major adverse cardiovascular event (MACE) and CV deaths in T2DM patients [27]. Combined with other previous studies [8, 12, 13, 26], we supposed that for older T2DM patients with baseline CV diseases or at high CV risk, TyG and cumulative TyG index may be useful parameters for risk stratification of death and MACE.

It was confirmed that TyG was a useful surrogate biochemical marker of IR [1, 2]. Because the calculation of TyG does not require insulin quantification, it is easily available in clinical practice and may be less influenced by insulin treatment. A recent study found that the TyG index had a better discrimination than HOMA-IR and HOMA-β for prediction of cardiovascular disease in a prospective large community-based cohort [28]. Results of another research based on the NHANES database also suggested that the discrimination value of TyG for albuminuria was higher than log(HOMA-IR) in a general population [29]. Our study was the first to evaluate the prognostic value of TyG in comparison to other IR indices in general T2DM patients. We found that based on the traditional risk factors, HOMA-IR, QUICKI, and HOMA-β had an equivalent discrimination as TyG did, even after we excluding patients with insulin treatment. Notably, the performance of TyG in predicting all-cause/non-CV death might be better than fasting glucose or triglyceride alone in T2DM patients younger than 65 years old.

There were several limitations in our study should be considered. First, we only used baseline TyG and could not assess how temporal changes in this biomarker may affect the association with cause-specific mortality. Several studies showed that cumulative TyG and visit-to-visit variability in TyG might be useful for the identification of individuals at high risk of CV events [17, 27] or diabetes [30]. Second, the current study did not have detailed information on the diabetes complication, although the results did not substantially change when further adjusting for comorbidities, diabetes medication use, and HbA1c levels. Third, this was a observational study so that some inherent bias such as unmeasured confounders could not be completely avoided. Fourth, more than 50% of the T2DM patients without baseline TyG were not included in our analyses. Compared with patients with TyG data, those without TyG had more heart failure, stroke and chronic kidney disease (lower eGFR), higher HbA1c and BMI, and more glucose lowering drugs. These differences may seriously limit the generalizability of our data to the NHANEs cohort as a whole. Therefore, studies are needed to confirm our findings in the future. Fifth, limited to the sample size, we did not assess the associations between TyG and cause-specific mortalities in older T2DM patients with different levels of HbA1c. In addition, the age cutoff in our study is selected empirically and at what age such associations would change needs to be explored. Last, only T2DM patients from the US were included in the study, thus generalizability to patients from other countries needs confirmation in the future.

## Conclusions

In a national sample of US patients with T2DM, we found that the associations between TyG and all-cause/non-CV death was modified by age. Higher TyG was associated with an increased risk of all-cause/non-CV only in T2DM patients younger than 65 years old, but not in older patients. Further studies are warranted to confirm these findings.

### Electronic supplementary material

Below is the link to the electronic supplementary material.

Supplementary Material 1

## Data Availability

The datasets generated and analysed during the current study are available in the NHANES repository (https://www.cdc.gov/nchs/nhanes/index.htm).

## Abbreviations

BMI: body mass index
CAD: coronary artery disease
CHF: congestive heart failure
CI: confidence interval
CKD: chronic kidney disease
CV: cardiovascular
eGFR: estimated glomerular filtration rate
HbA1c: glycated hemoglobin A1c
HDL-C: high density lipoprotein cholesterol
HOMA-IR: homeostasis model assessment of insulin resistance
HOMA-β: homeostasis model assessment of β-cell function
HR: hazard ratio
IR: insulin resistance
ICD-10: International Statistical Classification of Disease,10th Revision
LDL-C: low density lipoprotein cholesterol
NHANES: National Health and Nutrition Examination Survey
QUICKI: quantitative insulin sensitivity check index
RCS: restricted cubic spline
T2DM: type 2 diabetes mellitus
TyG: triglyceride-glucose
UA: uric acid
UACR: urine albumin-creatinine ratio
US: United States
