# Supplementary material for: The association between TyG and all-cause/non-cardiovascular mortality in general patients with type 2 diabetes mellitus is modified by age: results from the cohort study of NHANES 1999–2018
Source: Cardiovasc Diabetol. 2024 Jan 28;23:43. doi: 10.1186/s12933-024-02120-6 (PMC10823741; doi:10.1186/s12933-024-02120-6)
Supplement: Supplementary file 1 — Supplementary Material 1 [file 12933_2024_2120_MOESM1_ESM.docx]

**Supplemental Materials**

1. **Supplemental Tables**

**Supplemental Table 1. Cause of death in the total cohort**

**Supplemental Table 2. Baseline Characteristics of Patients with T2DM**

**Supplemental Table 3. Association between TyG and Cardiovascular Mortality**

**Supplemental Table 4. Association between TyG and Cancer Mortality**

**Supplemental Table 5. Predictive Power of Various Models with Different IR Indices (excluding patients with insulin treatments, Reference = TyG model)**

**Supplemental Table 6. Association between TyG and All-cause Mortality in Sensitivity Analysis**

**Supplemental Table 7. Association between TyG and Cardiovascular Mortality in Sensitivity Analysis**

**Supplemental Table 8. Association between TyG and Cancer Mortality in Sensitivity Analysis**

**Supplemental Table 9. Association between TyG and Non-cardiovascular Mortality in Sensitivity Analysis**

**2. Supplemental Figures**

**Supplemental Figure 1. Relationship between TyG and Other Insulin Resistance Indices by Age Group**

**Supplemental Figure 2. Association between TyG and Cardiovascular/Cancer Mortality**

**Supplemental Table 1. Cause of death in the total cohort**

| Cause of death | ICD-10 codes | Number of events |
| --- | --- | --- |
| All cause |  | 805 |
| Cardiovascular disease | I00-I09, I11, I13, and I20-I51 | 250 |
| Cancer | C00-C97 | 144 |
| Diabetes mellitus | E10-E14 | 80 |
| Alzheimer’s disease | G30 | 28 |
| Chronic lower respiratory diseases | J40-J47 | 25 |
| Nephritis, nephrotic syndrome, and nephrosis | N00-N07, N17-N19, and N25-N27 | 23 |
| Influenza and pneumonia | J09-J18 | 20 |
| Accidents (unintentional injuries) | V01-X59, Y85-Y86 | 16 |
| Other causes |  | 219 |

ICD, International Statistical Classification of Disease.

**Supplemental Table 2. Baseline Characteristics of Patients with T2DM**

| Variables | TyG < 8.82  (N=1,124) | 8.82≤TyG≤9.37  (N=1,124) | TyG > 9.37  (N=1,128) | P value |
| --- | --- | --- | --- | --- |
| Age (yrs) | 63 (53, 73) | 62.5 (52, 70) | 60 (50, 68) | <0.01 |
| Male | 580 (51.60%) | 563 (50.09%) | 607 (53.81%) | 0.206 |
| BMI (kg/m^2^) | 29.7 (25.8, 35.0) | 30.8 (26.9, 35.7) | 31.3 (27.6, 36.3) | < 0.001 |
| Ethnicity |  |  |  | < 0.001 |
| Mexican American | 154 (13.70%) | 228 (20.28%) | 310 (27.48%) |  |
| Non-Hispanic Black | 398 (35.41%) | 232 (20.64%) | 186 (16.49%) |  |
| Non-Hispanic White | 349 (31.05%) | 411 (36.57%) | 406 (35.99%) |  |
| Other Hispanic | 105 (9.34%) | 129 (11.48%) | 124 (10.99%) |  |
| Other Race | 118 (10.50%) | 124 (11.03%) | 102 (9.04%) |  |
| Educational level |  |  |  | 0.010 |
| Less than high school | 365 (32.47%) | 421 (37.46%) | 443 (39.27%) |  |
| High school or equivalent | 271 (24.11%) | 253 (22.51%) | 263 (23.32%) |  |
| College or above | 488 (43.42%) | 450 (40.04%) | 422 (37.41%) |  |
| Current smoking | 176 (15.66%) | 188 (16.73%) | 223 (19.77%) | 0.028 |
| **Comorbidities** |  |  |  |  |
| Congestive heart failure | 114 (10.14%) | 69 (6.14%) | 80 (7.09%) | 0.001 |
| Coronary artery disease | 177 (15.75%) | 150 (13.35%) | 152 (13.48%) | 0.185 |
| Hypertension | 901 (80.16%) | 911 (81.05%) | 866 (76.77%) | 0.030 |
| Stroke | 94 (8.36%) | 65 (5.78%) | 80 (7.09%) | 0.058 |
| CKD | 197 (17.5%) | 179 (15.9%) | 165 (14.6%) | 0.171 |
| **IR indices** |  |  |  |  |
| TyG | 8.47 (8.20, 8.67) | 9.10 (8.96, 9.23) | 9.78 (9.55, 10.08) | <0.001 |
| HOMA-IR | 3.07 (1.77, 5.25) | 4.75 (2.93, 7.28) | 7.46 (4.60, 12.65) | <0.001 |
| QUICKI | 0.32 (0.30, 0.35) | 0.30 (0.29, 0.33) | 0.29 (0.27, 0.31) | <0.001 |
| HOMA-β | 74.47  (41.61, 125.72) | 75.97  (43.61, 132.35) | 60.37  (30.29, 112.13) | < 0.001 |
| **Laboratory measurements** |  |  |  |  |
| HbA1c (%) | 6.3 (5.8, 6.8) | 6.5 (6.0, 7.3) | 7.4 (6.4, 9.2) | < 0.001 |
| Glucose (mg/dL) | 117 (103, 131) | 132 (118, 154) | 172 (137, 235) | < 0.001 |
| Triglyceride (mg/dL) | 79 (63, 96) | 131 (110, 155) | 211 (170, 261) | < 0.001 |
| Insulin (uU/mL) | 10.5 (6.4, 17.7) | 14.0 (8.9, 22.6) | 17.5 (10.4, 28.8) | < 0.001 |
| Albumin (g/dL) | 4.1 (3.9, 4.3) | 4.2 (3.9, 4.4) | 4.1 (3.9, 4.4) | < 0.001 |
| Serum creatinine (umol/L) | 78 (64, 96) | 75 (62, 90) | 73 (62, 90) | < 0.001 |
| eGFR (mL/1.73m^2^*min) | 87 (68, 102) | 87 (69, 102) | 91 (71, 104) | 0.002 |
| UACR (mg/g) | 10.3 (5.8, 31.7) | 11.5 (6.4, 36.2) | 17.8 (7.7, 55.6) | < 0.001 |
| UACR category |  |  |  | < 0.001 |
| <10 | 541 (48.1%) | 485 (43.2%) | 371 (32.9%) |  |
| 10 ~ <30 | 292 (26.0%) | 328 (29.2%) | 353 (31.3%) |  |
| 30 ~ <300 | 232 (20.6%) | 241 (21.4%) | 293 (26.0%) |  |
| ≥ 300 | 59 (5.3%) | 70 (6.2%) | 111 (9.8%) |  |
| Uric acid (umol/L) | 333.1  (279.6, 398.5) | 339 .0  (291.5, 398.5) | 339.0  (273.6, 404.5) | 0.266 |
| Total cholesterol (mmol/L) | 4.4 (3.8, 5.1) | 4.8 (4.1, 5.5) | 5.1 (4.4, 5.9) | < 0.001 |
| HDL-C (mmol/L) | 1.4 (1.2, 1.7) | 1.2 (1.1, 1.5) | 1.1 (0.9, 1.3) | < 0.001 |
| LDL-C (mmol/L) | 2.5 (1.9, 3.1) | 2.8 (2.2, 3.5) | 2.9 (2.3, 3.6) | < 0.001 |
| **Medications** |  |  |  |  |
| Glucose lowering drug | 606 (53.91%) | 618 (54.98%) | 678 (60.11%) | 0.007 |
| Insulin drug | 96 (8.54%) | 77 (6.85%) | 84 (7.45%) | 0.309 |
| Metformin | 303 (26.96%) | 316 (28.11%) | 305 (27.04%) | 0.790 |
| Anti-hypertension drug | 748 (66.55%) | 728 (64.77%) | 668 (59.22%) | 0.001 |
| Statins | 492 (43.77%) | 474 (42.17%) | 419 (37.15%) | 0.004 |
| Aspirin | 42 (3.74%) | 35 (3.11%) | 45 (3.99%) | 0.519 |
| Fibrates | 9 (0.80%) | 16 (1.42%) | 21 (1.86%) | 0.092 |

Values are number (percentage), or median (25th-75th percentile).

BMI, body mass index; CKD, chronic kidney disease; eGFR, estimated glomerular filtration rate; HbA1c, glucated hemoglobin; HDL-C, high-density-lipoprotein cholesterol; HOMA, homeostasis model assessment; IR, insulin resistance; LDL-C, low-density-lipoprotein cholesterol; QUICKI, quantitative insulin sensitivity check index; UACR, urine albumin-creatinine ratio.

**Supplemental Table 3. Association between TyG and Cardiovascular Mortality**

|  | Hazard ratio (95% confidence interval) | | | |
| --- | --- | --- | --- | --- |
|  | Model 1 | Model 2 | Model 3 | Model 4 |
| TyG (continuous) | P for interaction = 0.164 | P for interaction = 0.1759 | P for interaction = 0.105 | P for interaction = 0.331 |
| Total | 0.97 (0.80-1.18) | 1.10 (0.90-1.35) | 1.15 (0.93-1.41) | 0.91 (0.73-1.14) |
| < 65 yrs | 1.27 (0.90-1.78) | 1.28 (0.90-1.83) | 1.28 (0.91-1.80) | 0.94 (0.62-1.42) |
| ≥ 65 yrs | 0.97 (0.76-1.24) | 1.03 (0.80-1.31) | 1.08 (0.84-1.39) | 0.90 (0.68-1.19) |
| TyG (categorical) | P for interaction = 0.031 | P for interaction = 0.033 | P for interaction = 0.021 | P for interaction = 0.059 |
| TyG tertile 1 | Reference | Reference | Reference | Reference |
| Total |  |  |  |  |
| TyG tertile 2 | 0.78 (0.57-1.07) | 0.80 (0.58-1.09) | 0.90 (0.65-1.23) | 0.81 (0.58-1.11) |
| TyG tertile 3 | 0.91 (0.67-1.22) | 1.07 (0.79-1.44) | 1.17 (0.86-1.60) | 0.86 (0.61-1.21) |
| < 65 yrs |  |  |  |  |
| TyG tertile 2 | 1.04 (0.53-2.01) | 0.99 (0.51-1.92) | 1.16 (0.59-2.29) | 1.06 (0.53-2.14) |
| TyG tertile 3 | 1.61 (0.89-2.89) | 1.60 (0.89-2.89) | 1.73 (0.94-3.18) | 1.28 (0.65-2.55) |
| ≥ 65 yrs |  |  |  |  |
| TyG tertile 2 | **0.69 (0.48-0.99)*** | 0.80 (0.56-1.15) | 0.88 (0.61-1.27) | 0.79 (0.54-1.14) |
| TyG tertile 3 | 0.83 (0.58-1.19) | 0.91 (0.64-1.31) | 1.00 (0.69-1.45) | 0.75 (0.50-1.13) |

Model 1 was unadjusted.

Model 2 was adjusted for age and gender.

Model 3 was adjusted for age, gender, ethnicity, CHF, CAD, albumin, eGFR, and insulin treatment.

Model 4 was adjusted for age, gender, ethnicity, educational level, current smoking, BMI, CHF, CAD, hypertension, albumin, LDL-C, eGFR, HbA1c, UACR, UA, Insulin treatment, metformin, and statins.

* indicated that the p value was less than 0.05.

**Supplemental Table 4. Association between TyG and Cancer Mortality**

|  | Hazard ratio (95% confidence interval) | | | |
| --- | --- | --- | --- | --- |
|  | Model 1 | Model 2 | Model 3 | Model 4 |
| TyG (continuous) | P for interaction = 0.089 | P for interaction = 0.100 | P for interaction = 0.117 | P for interaction = 0.048 |
| Total | 0.82 (0.64-1.05) | 0.89 (0.68-1.16) | 0.89 (0.68-1.16) | 0.89 (0.66-1.20) |
| < 65 yrs | 1.16 (0.75-1.79) | 1.18 (0.75-1.85) | 1.16 (0.74-1.82) | 1.23 (0.70-2.15) |
| ≥ 65 yrs | 0.74 (0.53-1.02) | 0.76 (0.55-1.06) | 0.77 (0.55-1.07) | 0.78 (0.55-1.11) |
| TyG (categorical) | P for interaction = 0.426 | P for interaction = 0.441 | P for interaction = 0.505 | P for interaction = 0.305 |
| TyG tertile 1 | Reference | Reference | Reference | Reference |
| Total |  |  |  |  |
| TyG tertile 2 | 0.78 (0.53-1.17) | 0.79 (0.53-1.18) | 0.79 (0.53-1.18) | 0.82 (0.54-1.24) |
| TyG tertile 3 | 0.77 (0.52-1.14) | 0.88 (0.59-1.31) | 0.89 (0.60-1.32) | 0.90 (0.58-1.41) |
| < 65 yrs |  |  |  |  |
| TyG tertile 2 | 0.87 (0.40-1.88) | 0.83 (0.39-1.80) | 0.83 (0.38-1.79) | 0.75 (0.33-1.67) |
| TyG tertile 3 | 1.03 (0.51-2.08) | 1.04 (0.51-2.10) | 1.02 (0.50-2.07) | 1.02 (0.45-2.27) |
| ≥ 65 yrs |  |  |  |  |
| TyG tertile 2 | 0.71 (0.44-1.15) | 0.77 (0.48-1.24) | 0.77 (0.48-1.24) | 0.84 (0.51-1.38) |
| TyG tertile 3 | 0.76 (0.46-1.24) | 0.81 (0.49-1.32) | 0.82 (0.50-1.34) | 0.86 (0.50-1.48) |

Model 1 was unadjusted.

Model 2 was adjusted for age and gender.

Model 3 was adjusted for age, gender, and CAD.

Model 4 Model 4 was adjusted for age, gender, ethnicity, educational level, current smoking, BMI, CHF, CAD, hypertension, albumin, LDL-C, eGFR, HbA1c, UACR, UA, Insulin treatment, metformin, and statins.

**Supplemental Table 5. Predictive Power of Various Models with Different IR Indices (excluding patients with insulin treatments, Reference = TyG model)**

| Base model | + TyG | + HOMA-IR |  | + QUICKI |  | + HOMA-beta |  | + Glucose |  | + Triglyceride |  |
| --- | --- | --- | --- | --- | --- | --- | --- | --- | --- | --- | --- |
|  | C-index  (95% CI) | C-index  (95% CI) | P value | C-index  (95% CI) | P value | C-index  (95% CI) | P value | C-index  (95% CI) | P value | C-index  (95% CI) | P value |
| All-cause mortality | | | | | | | |  |  |  |  |
| Total | 0.79 (0.78-0.81) | 0.79  (0.78-0.81) | 0.368 | 0.79  (0.78-0.81) | 0.248 | 0.79  (0.78-0.81) | 0.107 | 0.80  (0.78-0.81) | 0.788 | 0.79  (0.78-0.81) | 0.359 |
| < 65 yrs | 0.75 (0.72-0.79) | 0.74  (0.70-0.78) | 0.266 | 0.74  (0.70-0.78) | 0.112 | 0.73  (0.69-0.77) | 0.052 | 0.75  (0.71-0.79) | 0.920 | 0.74  (0.70-0.78) | **0.028** |
| ≥ 65 yrs | 0.74 (0.72-0.76) | 0.74  (0.72-0.76) | 0.731 | 0.74  (0.72-0.76) | 0.897 | 0.74  (0.72-0.76) | 0.944 | 0.74  (0.72-0.76) | 0.689 | 0.74  (0.72-0.76) | 0.787 |
| Cardiovascular mortality | | | | | | | |  |  |  |  |
| Total | 0.82  (0.79-0.85) | 0.82  (0.79- 0.85) | 0.137 | 0.82  (0.79- 0.85) | 0.189 | 0.82  (0.79- 0.85) | 0.161 | 0.82  (0.79-0.85) | 0.832 | 0.82  (0.79-0.85)) | 0.111 |
| < 65 yrs | 0.83  (0.76-0.89) | 0.82  (0.75-0.89) | 0.476 | 0.81  (0.74-0.89) | 0.373 | 0.82  (0.75- 0.89) | 0.469 | 0.83  (0.77-0.90) | 0.320 | 0.82  (0.75-0.88) | 0.281 |
| ≥ 65 yrs | 0.76  (0.72- 0.80) | 0.76  (0.72- 0.80) | 0.879 | 0.77  (0.73- 0.81) | 0.583 | 0.77  (0.73-0.81) | 0.545 | 0.76  (0.72-0.80) | 0.690 | 0.76  (0.72-0.80) | 0.447 |
| Cancer mortality | | | | | | | |  |  |  |  |
| Total | 0.74  (0.70-0.79) | 0.74  (0.70-0.79) | 0.638 | 0.74  (0.70-0.79) | 0.860 | 0.74  (0.70-0.79) | 0.704 | 0.74  (0.70-0.79) | 0.608 | 0.74  (0.70-0.79) | 0.721 |
| < 65 yrs | 0.68  (0.59-0.77) | 0.68  (0.59-0.76) | 0.728 | 0.69  (0.60-0.77) | 0.884 | 0.68  (0.59-0.76) | 0.710 | 0.68  (0.60-0.76) | 0.948 | 0.69  (0.60-0.78) | 0.203 |
| ≥ 65 yrs | 0.63  (0.57-0.70 ) | 0.64  (0.58-0.70) | 0.603 | 0.64  (0.57-0.70) | 0.725 | 0.64  (0.57-0.70) | 0.702 | 0.64  (0.57-0.70) | 0.751 | 0.63  (0.57-0.70) | 0.904 |
| Non-cardiovascular mortality | | | | | | | |  |  |  |  |
| Total | 0.79  (0.77-0.81) | 0.79  (0.77-0.81) | 0.315 | 0.79  (0.76-0.81) | 0.405 | 0.79  (0.76-0.81) | 0.431 | 0.79  (0.77-0.81 | 0.349 | 0.79  (0.76-0.81) | 0.555 |
| < 65 yrs | 0.73  (0.68-0.77) | 0.72  (0.67-0.76) | 0.540 | 0.71  (0.66-0.76) | 0.291 | 0.71  (0.66-0.75) | 0.115 | 0.72  (0.68-0.76) | 0.493 | 0.72  (0.66-0.75) | **0.048** |
| ≥ 65 yrs | 0.74  (0.71-0.76) | 0.74  (0.71-0.77) | 0.160 | 0.74  (0.76-0.76) | 0.806 | 0.74  (0.71-0.76) | 0.764 | 0.74  (0.71-0.76) | 0.446 | 0.74  (0.71-0.76) | 0.859 |

Base models included the covariates which were selected by the stepwise method in Model 3.

**Supplemental Table 6. Association between TyG and All-cause Mortality in Sensitivity Analysis**

|  | Without CHF | Without CAD | Without stroke | eGFR > 60 mL/(1.73m2*min) | Without insulin treatment |
| --- | --- | --- | --- | --- | --- |
| TyG (continuous) | P for interaction < 0.001 | P for interaction <0.001 | P for interaction < 0.001 | P for interaction <0.001 | P for interaction < 0.001 |
| Total | 1.09 (0.95-1.25) | 1.04 (0.89-1.20) | 1.02 (0.90-1.17) | 1.03 (0.89-1.22) | 1.08 (0.94-1.25) |
| < 65 yrs | **1.36 (1.06-1.75)*** | **1.35 (1.04-1.76)*** | **1.33 (1.05-1.68)*** | **1.40 (1.09-1.80)*** | **1.39 (1.03-1.81)*** |
| ≥ 65 yrs | 1.00 (0.84-1.19) | 0.96 (0.80-1.15) | 0.94 (0.80-1.12) | 0.87 (0.70-1.07) | 1.01 (0.86-1.20) |
| TyG (categorical) | P for interaction < 0.001 | P for interaction < 0.001 | P for interaction < 0.001 | P for interaction <0.001 | P for interaction < 0.001 |
| TyG tertile 1 | reference | reference | reference | reference | reference |
| Total |  |  |  |  |  |
| TyG tertile 2 | 0.90 (0.73-1.10) | 0.86 (0.69-1.07) | 0.86 (0.71-1.04) | 0.81 (0.65-1.01) | 0.87 (0.72-1.06) |
| TyG tertile 3 | 1.12 (0.91-1.38) | 1.00 (0.80-1.25) | 1.02 (0.83-1.24) | 0.91 (0.72-1.15) | 1.04 (0.85-1.27) |
| < 65 yrs |  |  |  |  |  |
| TyG tertile 2 | 1.06 (0.70-1.61) | 1.08 (0.70-1.67) | 1.03 (0.69-1.53) | 1.07 (0.72-1.60) | 1.01 (0.67-1.52) |
| TyG tertile 3 | **1.54 (1.04-2.29)*** | 1.51 (0.99-2.29) | **1.49 (1.02-2.16)*** | **1.66 (1.12-2.48)*** | **1.54 (1.03-2.31)*** |
| ≥ 65 yrs |  |  |  |  |  |
| TyG tertile 2 | 0.91 (0.72-1.16) | 0.87 (0.67-1.12) | 0.86 (0.68-1.08) | 0.76 (0.57-1.02) | 0.90 (0.72-1.13) |
| TyG tertile 3 | 1.02 (0.79-1.32) | 0.90 (0.69-1.28) | 0.92 (0.72-1.18) | 0.72 (0.53-1.00) | 0.94 (0.74-1.19) |

Models were adjusted for age, gender, ethnicity, educational level, current smoking, BMI, CHF, CAD, hypertension, albumin, LDL-C, eGFR, HbA1c, UACR, UA, Insulin treatment, metformin, and statins.

* indicated that the p value was less than 0.05.

**Supplemental Table 7. Association between TyG and Cardiovascular Mortality in Sensitivity Analysis**

|  | Without CHF | Without CAD | Without stroke | eGFR > 60 mL/(1.73m2*min) | Without insulin treatment |
| --- | --- | --- | --- | --- | --- |
| TyG (continuous) | P for interaction = 0.932 | P for interaction = 0.738 | P for interaction = 0.456 | P for interaction = 0.141 | P for interaction = 0.166 |
| Total | 1.01 (0.78-1.32) | 0.89 (0.67-1.18) | 0.91 (0.72-1.16) | 1.02 (0.77-1.36) | 1.11 (0.87-1.43) |
| < 65 yrs | 0.79 (0.49-1.26) | 0.64 (0.38-1.08) | 0.88 (0.57-1.37) | 1.14 (0.72-1.82) | 1.27 (0.79-2.07) |
| ≥ 65 yrs | 1.17 (0.84-1.63) | 1.09 (0.78-1.52) | 0.95 (0.71-1.27) | 0.98 (0.66-1.44) | 1.08 (0.80-1.46) |
| TyG (categorical) | P for interaction = 0.341 | P for interaction = 0.346 | P for interaction = 0.068 | P for interaction = 0.013 | P for interaction = 0.019 |
| TyG tertile 1 | reference | reference | reference | reference | reference |
| Total |  |  |  |  |  |
| TyG tertile 2 | 0.92 (0.63-1.33) | 0.89 (0.60-1.31) | 0.83 (0.59-1.17) | 0.94 (0.62-1.42) | 0.91 (0.64-1.30) |
| TyG tertile 3 | 1.01 (0.68-1.51) | 0.79 (0.52-1.21) | 0.89 (0.62-1.28) | 0.92 (0.59-1.45) | 1.04 (0.72-1.50) |
| < 65 yrs |  |  |  |  |  |
| TyG tertile 2 | 0.95 (0.45-2.03) | 1.12 (0.50-2.51) | 1.12 (0.53-2.35) | 1.53 (0.69-3.38) | 1.39 (0.62-3.12) |
| TyG tertile 3 | 1.15 (0.54-2.42) | 0.88 (0.37-2.05) | 1.31 (0.63-2.74) | 1.69 (0.74-3.83) | 1.99 (0.89-4.47) |
| ≥ 65 yrs |  |  |  |  |  |
| TyG tertile 2 | 1.04 (0.68-1.60) | 0.94 (0.59-1.48) | 0.80 (0.54-1.19) | 0.87 (0.52-1.46) | 0.91 (0.61-1.36) |
| TyG tertile 3 | 1.05 (0.65-1.70) | 0.85 (0.52-1.38) | 0.80 (0.52-1.23) | 0.69 (0.38-1.26) | 0.87 (0.56-1.34) |

Models were adjusted for age, gender, ethnicity, educational level, current smoking, BMI, CHF, CAD, hypertension, albumin, LDL-C, eGFR, HbA1c, UACR, UA, Insulin treatment, metformin, and statins.

**Supplemental Table 8. Association between TyG and Cancer Mortality in Sensitivity Analysis**

|  | Without CHF | Without CAD | Without stroke | eGFR > 60 mL/(1.73m2*min) | Without insulin treatment |
| --- | --- | --- | --- | --- | --- |
| TyG (continuous) | P for interaction = 0.060 | P for interaction = 0.021 | P for interaction = 0.033 | P for interaction = 0.196 | P for interaction = 0.114 |
| Total | 0.94 (0.69-1.30) | 0.91 (0.65-1.27) | 0.91 (0.67-1.24) | 1.00 (0.70-1.40) | 0.86 (0.63-1.19) |
| < 65 yrs | 1.40 (0.76-2.60) | 1.45 (0.76-2.76) | 1.21 (0.69-2.11) | 1.30 (0.72-2.35) | 1.12 (0.61-2.06) |
| ≥ 65 yrs | 0.82 (0.56-1.19) | 0.76 (0.51-1.13) | 0.80 (0.55-1.17) | 0.83 (0.54-1.29) | 0.78 (0.54-1.14) |
| TyG (categorical) | P for interaction = 0.178 | P for interaction = 0.098 | P for interaction = 0.232 | P for interaction = 0.676 | P for interaction = 0.396 |
| TyG tertile 1 | reference | reference | reference | reference | reference |
| Total |  |  |  |  |  |
| TyG tertile 2 | 0.83 (0.53-1.30) | 0.71 (0.44-1.15) | 0.80 (0.52-1.24) | 0.79 (0.48-1.30) | 0.75 (0.48-1.17) |
| TyG tertile 3 | 1.08 (0.67-1.72) | 0.88 (0.53-1.44) | 0.91 (0.57-1.45) | 1.00 (0.59-1.67) | 0.90 (0.57-1.42) |
| < 65 yrs |  |  |  |  |  |
| TyG tertile 2 | 1.00 (0.41-2.48) | 0.73 (0.29-1.84) | 0.72 (0.32-1.61) | 0.81 (0.36-1.85) | 0.62 (0.27-1.47) |
| TyG tertile 3 | 1.51 (0.62-3.71) | 1.31 (0.53-3.27) | 1.00 (0.45-2.22) | 1.08 (0.46-2.53) | 0.95 (0.40-2.21) |
| ≥ 65 yrs |  |  |  |  |  |
| TyG tertile 2 | 0.78 (0.46-1.32) | 0.75 (0.42-1.35) | 0.86 (0.51-1.45) | 0.75 (0.39-1.42) | 0.81 (0.48-1.36) |
| TyG tertile 3 | 0.96 (0.54-1.69) | 0.75 (0.40-1.40) | 0.89 (0.50-1.60) | 0.95 (0.49-1.85) | 0.86 (0.49-1.51) |

Models were adjusted for age, gender, ethnicity, educational level, current smoking, BMI, CHF, CAD, hypertension, albumin, LDL-C, eGFR, HbA1c, UACR, UA, Insulin treatment, metformin, and statins.

**Supplemental Table 9. Association between TyG and Non-cardiovascular Mortality in Sensitivity Analysis**

|  | Without CHF | Without CAD | Without stroke | Without CKD | Without insulin treatment |
| --- | --- | --- | --- | --- | --- |
| TyG (continuous) | P for interaction < 0.001 | P for interaction < 0.001 | P for interaction < 0.001 | P for interaction < 0.001 | P for interaction < 0.001 |
| Total | 1.12 (0.95-1.33) | 1.11 (0.93-1.32) | 1.09 (0.93-1.28) | 1.05 (0.87-1.27) | 1.08 (0.91-1.27) |
| < 65 yrs | **1.68 (1.25-2.26)*** | **1.74 (1.27-2.38)*** | **1.58 (1.19-2.08)*** | **1.53 (1.13-2.06)*** | **1.46 (1.06-2.01)*** |
| ≥ 65 yrs | 0.95 (0.77-1.18) | 0.93 (0.74-1.16) | 0.95 (0.77-1.17) | 0.83 (0.64-1.07) | 0.99 (0.81-1.22) |
| TyG (categorical) | P for interaction < 0.001 | P for interaction < 0.001 | P for interaction < 0.001 | P for interaction < 0.001 | P for interaction = 0.005 |
| TyG tertile 1 | reference | reference | reference | reference | reference |
| Total |  |  |  |  |  |
| TyG tertile 2 | 0.89 (0.70-1.13) | 0.86 (0.66-1.11) | 0.87 (0.69-1.10) | 0.76 (0.58-1.00) | 0.86 (0.68-1.08) |
| TyG tertile 3 | 1.17 (0.92-1.49) | 1.10 (0.85-1.43) | 1.09 (0.85-1.38) | 0.91 (0.69-1.20) | 1.05 (0.83-1.33) |
| < 65 yrs |  |  |  |  |  |
| TyG tertile 2 | 1.12 (0.69-1.84) | 1.06 (0.64-1.78) | 0.99 (0.62-1.59) | 1.01 (0.63-1.61) | 0.90 (0.55-1.45) |
| TyG tertile 3 | **1.77 (1.11-2.83)*** | **1.77 (1.09-2.87)*** | **1.56 (1.00-2.41)*** | 1.57 (0.99-2.49) | 1.42 (0.89-2.27) |
| ≥ 65 yrs |  |  |  |  |  |
| TyG tertile 2 | 0.88 (0.67-1.16) | 0.86 (0.63-1.17) | 0.89 (0.68-1.18) | 0.73 (0.52-1.03) | 0.90 (0.69-1.19) |
| TyG tertile 3 | 1.03 (0.76-1.40) | 0.95 (0.69-1.31) | 1.00 (0.74-1.35) | 0.75 (0.51-1.09) | 0.99 (0.74-1.32) |

Models were adjusted for age, gender, ethnicity, educational level, current smoking, BMI, CHF, CAD, hypertension, albumin, LDL-C, eGFR, HbA1c, UACR, UA, Insulin treatment, metformin, and statins.

* indicated that the p value was less than 0.05.

**Supplemental Figure 1. Relationship between TyG and Other Insulin Resistance Indices by Age Group**

**
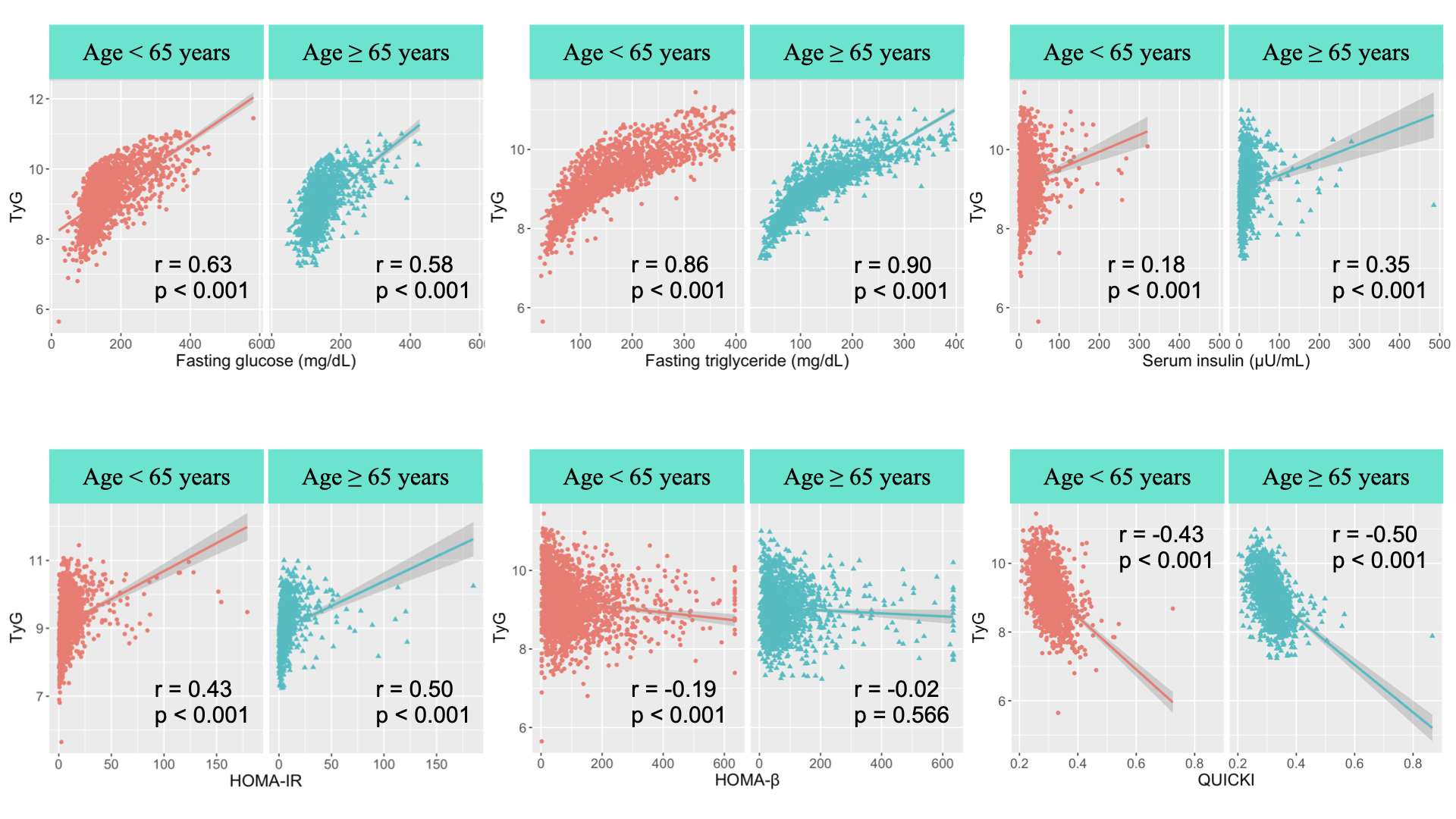
**

**Supplemental Figure 2. Association between TyG and Cardiovascular/Cancer Mortality**

**
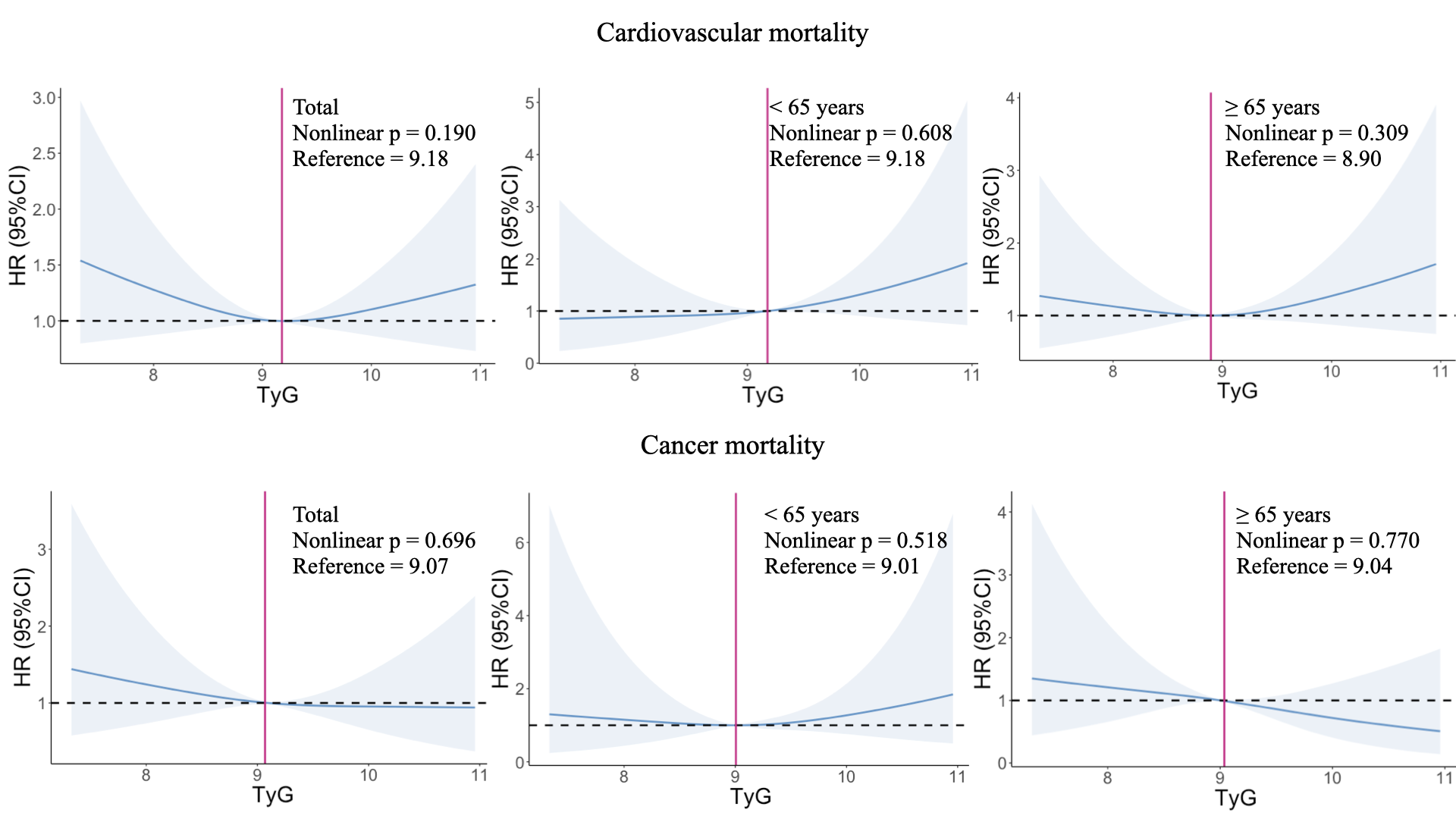
**

Models for cardiovascular mortality were adjusted for age, ethnicity, CHF, CAD, albumin, eGFR, and insulin treatment. There was no significant association between TyG and cardiovascular death in total cohort, younger group, and older group.

Models for cancer mortality were adjusted for age, gender, and CAD. There was no significant association between TyG and cancer death in total cohort, younger group, and older group.
